# Matthew Large

**DOI:** 10.1192/bjb.2020.149

**Published:** 2021-06

**Authors:** Abdi Sanati

**Figure:**
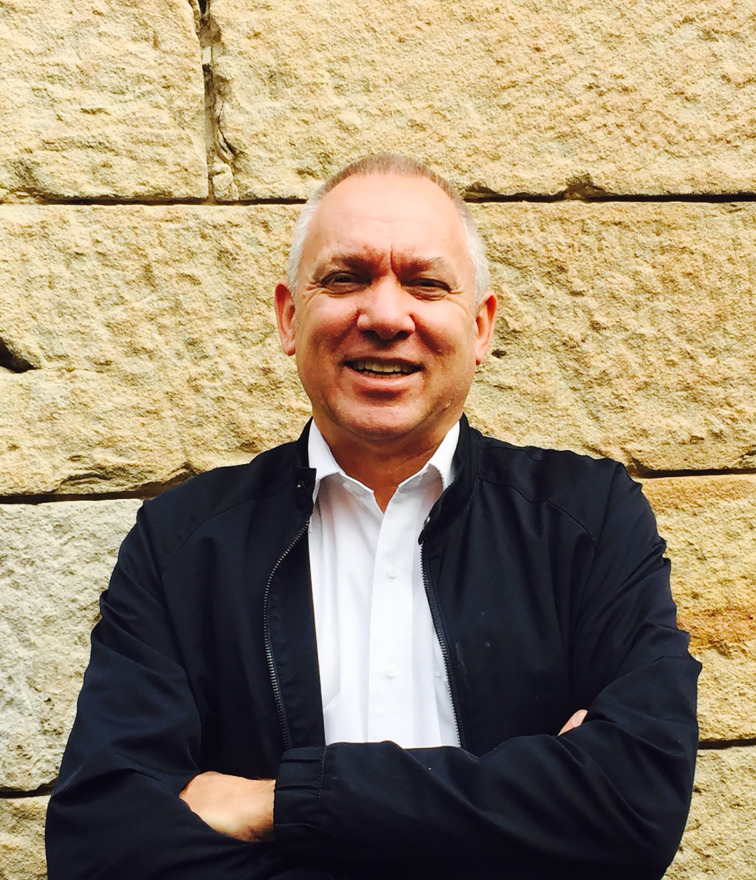

As psychiatrists, we engage with risk assessment on a daily basis. I personally have seen an increased emphasis on risk assessment since I started training two decades ago. The question for me has always been how accurate our risk assessments are. I then read the works of Professor Matthew Large and was heartened by his common-sense approach to risk. We met a few years ago in a Maudsley debate on risk. Professor Large has written extensively on risk assessment in psychiatry. Although he holds an academic title, he works in emergency departments and acute wards. He knows the reality of working as a psychiatrist. He lives and works in Sydney, Australia.

**Thank you very much for your time, Professor Large. I wanted to start by asking what do you think the place of risk assessment is in psychiatry?**

Before answering, I should say a few things. People who read my papers sometimes think of me as a cloistered academic, but in Australia I am a full-time clinician. I mostly work in emergency rooms and observation wards. In the past 2 days I have seen 21 hospital patients, quite a few of whom were new to me. In the Maudsley debate I was criticised for being detached from the reality of day-to-day work, but the opposite is the truth. Perhaps the most fundamental criticism of risk assessment relates to the very nature of day-to-day psychiatric practice. I don't think any of us really work with low-risk patients – I am at a loss to think who these patients even are. Therefore, in our work, risk assessment must have a modest role. Nevertheless, I acknowledge that we can moderately differentiate between people at higher risk and lower risk. My main criticism of risk assessment is not that it gives you no information, but that the amount of information it provides is so slight. The vast majority of people classified as higher risk don't engage in seriously harmful behaviour and in most studies about half of the seriously harmful events occurred in lower-risk people. Taking this one step forward, if you can imagine an intervention that is effective, benign and acceptable to high-risk patients (the vast majority of whom will never experience serious harm), how can such an intervention be rationally denied to lower-risk patients, among whom half of all serious harm events occur?

**I remember reading about a paper by Frank Knight published in 1922, where he distinguishes risk from uncertainty, the former being quantifiable, the latter not. The paper was in the context of insurance, but don't you think it has a relevance in the practice of risk assessment in psychiatry?**

Uncertainty is a huge driver of medical diagnostics but we do not think about it enough. As a concept uncertainty is more fruitful than risk in psychiatry. Consider the following scenario. A 16-year-old smoker may or may not develop lung cancer. Here the uncertainty is mostly due to chance and more information cannot really help you. Aged 50 the same smoker might develop haemoptysis. Whether or not the person has lung cancer is now a matter of discernible knowledge. These two types of uncertainty, sometimes called aleatory and epistemic uncertainty, are often confused. For example, while we might establish with some degree of certainty that a patient has suicidal ideas, this means surprisingly little about the chance of later suicide. More bluntly, while our risk assessments can tell us something about the patient in front of us, they are not a meaningful prediction. Unfortunately, this is not always understood and can and does have bad consequences for patients. I started thinking about this in relation to mental health law. When a group of patients are detained on the basis of a perceived risk, all higher-risk patients, including the majority false-positive group, share the burden of the resulting interventions. This is actually very old-fashioned collectivism and most of us would much prefer to carry our own risk if we were ever in this situation.

**Interesting that you mentioned mental health law. I remember you wrote about the role of the dangerousness criterion. Could this criterion be useful in people like a patient who attacks his mother when he is unwell?**

Sadly, we are stuck with the term dangerousness. To answer your question, if you had a patient who you knew attacks his mother when he is unwell this could be considered to be an epistemic dimension for him and might contribute to a treatment decision. However, I think he could be treated when he is so unwell as to attack his mother and because of a lack of mental capacity to refuse treatment. No recourse to risk is actually required. Further, this sort of specific knowledge about one patient is hard to generalise to others. Research invariably shows that if you do generalise a violence risk factor this will only modestly differentiate between higher- and lower-risk patients. Even when risk factors are combined, risk assessment does not work very well – a recent meta-analysis by Seena Fazel's group found that the odds ratio (OR) for violence among higher-risk patients compared with lower-risk patients was 6 under optimal research conditions and 3 under more ordinary circumstances. An OR of 6 sounds high but remember that being male is associated with an OR for homicide of about 10 and an OR for suicide of 4 in most countries. What differentiates maleness from psychiatric risk factors is the negative value or stigma associated with mental illness.

**You recently co-authored a study on the relationship between suicidal ideation and later suicide. What intrigued me was that there was not much difference between the predictive power of risk assessment based on clinical examination and risk assessment based on actuarial tools.**

I accept the work of Paul Meehl that found that actuarial assessments are generally a bit more accurate than clinical assessments. What this does not mean is that actuarial assessments are sufficiently good to be a basis for clinical decisions. At least a clinical risk assessment opens some possibility for engagement with the patient and consideration of their unique qualities and needs. If used sensibly, a clinical assessment might even lead to a meaningful or helpful dialogue. A tick-box approach demeans the assessed person's human agency and decision-making and likely does much the same to the risk assessor. Patients have every bit as much human agency as we do. I always try to assess the extent to which my patients are risk assessing me as someone who can harm them by depriving them of their liberty and by enforcing treatments. I am eager to see game theory applied to risk assessment – I think there might be some fruitful work to be done in this area.

**One striking feature of risk assessment tools is their poor positive predictive value (PPV). I wonder how you think tests with such a low PPV could help clinical decisions?**

Well, I don't think they can. Any test with such a high number of false positives is pretty useless in medicine. I try to focus on clinical needs. Sometimes needs converge with risk but risk rarely tips my decisions. An excessive focus on risk can contribute to unnecessary hospitalisation. Psychiatrists and the media (particularly in the UK) often focus on the very rare event of a homicide by a patient with schizophrenia. My colleagues and I did a couple of studies some years ago and that found that 35 000 patients would need to be detained to prevent one such stranger homicide. Tangentially to this, what we worry about is largely a question of our value systems. I have recently been focusing on the relative risks of suicide and vascular death after discharge from psychiatric hospital: in reality, the risk of vascular death exceeds suicide within months of discharge, yet worries us much less.

**There is also the role of politics. A few years ago, the UK deputy prime minister spoke of zero suicide and the focus was mainly on mental health services. Could it be that by putting all the responsibility of suicide prevention on mental health services, the politicians avoid the social changes that are necessary for reducing suicide? Changes such as reducing inequality, helping families, providing meaningful employment, among others.**

Let's look at this objectively. The prevalence of mental illnesses does not vary that much between countries but the incidence of suicide varies dramatically. We have made a rod for our own backs by believing that 90% of suicides are *because* of mental illness. While mental illness is associated with suicide, this is not always causal and mental illness and suicide have many common underlying causes, for example social disadvantage, stigma and substance use. Even if we assume a big role for mental illness in suicide, where does this lead us? With the possible exceptions of lithium and clozapine, there is pretty much no evidence that psychopharmacological treatments reduce suicide, and suicide rates in hospital and aftercare are very high, suggesting we are not good at protecting our patients.

**In my own experience, I have witnessed some divergence between academics and clinicians. I remember once in a debate an academic quoting research, which I add was not designed to investigate suicidality, to say all suicide was because of mental illness and no one can rationally contemplate suicide.**

There is a divide. The question I have is who is a ‘suicidal patient’? It is almost insulting to think that the process of listening to patients and observing their unique or idiographic characteristics can be reduced to a few common or nomothetic risk factors. I think risk assessment should be replaced by risk communication – a communication that we must have with patients and their families. We should be open about the uncertainties and the low power of our predictive tools. We need to be honest about our limitations.

**One problem that we face is that the inaccuracy of risk assessment tools has to be communicated to the courts, judges and coroners. How do you think we can do it?**

The courts can get this very wrong. In the case of Melanie Rabone^a^ the Supreme Court heard expert psychiatric evidence that overestimated suicide risk by two orders of magnitude. We need to explain to the courts that the presence of multiple and statistically valid risk factors does not equal useful knowledge about the future and that we are doctors and not soothsayers.

## Footnotes

a. In an important judgment in 2012 concerning the suicide of Melanie Rabone, a 24-year-old woman who was an informal patient, the UK Supreme Court ruled that the state has a duty to all patients under Article 2 of the European Convention on Human Rights. For further information see: https://www.supremecourt.uk/cases/docs/uksc-2010-0140-judgment.pdf.

